# The gynoecious CmWIP1 transcription factor interacts with CmbZIP48 to inhibit carpel development

**DOI:** 10.1038/s41598-019-52004-z

**Published:** 2019-10-28

**Authors:** John S. Y. Eleblu, Aimen Haraghi, Brahim Mania, Celine Camps, Dali Rashid, Halima Morin, Catherine Dogimont, Adnane Boualem, Abdelhafid Bendahmane

**Affiliations:** 1 Institute of Plant Sciences Paris-Saclay IPS2, CNRS, INRA, University Paris-Sud, University of Evry, University Paris-Diderot, University of Paris-Saclay, Orsay, France; 2 UR 1052, Unité de Génétique et d’Amélioration des Fruits et Légumes, INRA, BP94, Montfavet, France

**Keywords:** Plant development, Plant reproduction

## Abstract

In angiosperms, sex determination leads to development of unisexual flowers. In *Cucumis melo*, development of unisexual male flowers results from the expression of the sex determination gene, *CmWIP1*, in carpel primordia. To bring new insight on the molecular mechanisms through which *CmWIP1* leads to carpel abortion in male flowers, we used the yeast two-hybrid approach to look for CmWIP1-interacting proteins. We found that CmWIP1 physically interacts with an S2 bZIP transcription factor, CmbZIP48. We further determined the region mediating the interaction and showed that it involves the N-terminal part of CmWIP1. Using laser capture microdissection coupled with quantitative real-time gene expression analysis, we demonstrated that *CmWIP1* and *CmbZIP48* share a similar spatiotemporal expression pattern, providing the plant organ context for the CmWIP1-CmbZIP48 protein interaction. Using sex transition mutants, we demonstrated that the expression of the male promoting gene *CmWIP1* correlates with the expression of *CmbZIP48*. Altogether, our data support a model in which the coexpression and the physical interaction of CmWIP1 and CmbZIP48 trigger carpel primordia abortion, leading to the development of unisexual male flowers.

## Introduction

Most flowering plants are hermaphroditic, producing only bisexual flowers. Sex determination is a developmental process that leads to unisexual flowers. Beside hermaphrodite plants, 10% of the species display unisexual flowers. Monoecious species such as *Cucumis melo*, here after melon, exhibit male and female flowers on the same plant. Dioecious species, such as Asparagus and date palm, have separate male and female individuals^1,2^.

In melon, flowers are bisexual at early developmental stages then sex determination occurs by the developmental arrest of either the stamen or the carpel primordia, resulting in unisexual flowers. This sexual organ arrest is genetically governed by the interaction of the *andromonoecious* (*M*), *androecious* gene (*A*) and *gynoecious* (*G*) genes that define the monoecy sex determination pathway in cucurbits^3^. Genetic analysis revealed a mechanistic model in which expression of the carpel inhibitor, *G* gene, is dependent on non-expression of *A* gene and expression of the stamina inhibitor, *M* gene, is dependent on non-expression of *G* gene.

The cloning and characterization of the three sex determination genes revealed the identity of *M*, *A* and *G* genes as *CmACS-7*, *CmACS11* and *CmWIP1*, respectively. *CmACS-7* and *CmACS11* encode for aminocyclopropane-1-carboxylic acid synthases, an enzyme that catalyse the rate limiting step of ethylene biosynthesis in plants. *CmWIP1* belongs to a class of C_2_H_2_ zinc finger transcription factors named WIP proteins, based on the presence of a conserved first 3 C-terminal amino acids, WIP^4,5^. Investigation of sex determination in other cucurbits, including cucumber and watermelon revealed a conserved sex determination pathway that implicates *CmACS-7*, *CmACS11* and *CmWIP1* homologs^3,6–8^.

While the molecular mechanisms through which *CmACS-7* and *CmACS11* control sex determination may be predicted, how *CmWIP1* contributes to carpel inhibition is an enigma. In *Arabidopsis thaliana*, six WIP proteins have been identified and through mutation analysis WIP proteins were associated with seed coat development (*AtWIP1*)^4^, transmitting tract growth (*AtWIP2*)^9^, leaf vein patterning (*AtWIP6*)^10^ and in the early root apical meristem formation^11^. A common theme and character demonstrated by the WIP zinc finger proteins points to a regulatory role of these genes in organ growth and development. This hypothesis has been further validated by a recent study demonstrating that in Arabidopsis, the *ntt/wip4/wip5* triple mutants fail to develop roots^11^.

BLAST search analysis revealed that WIP proteins are restricted to land plants, including the bryophyte *Physcomitrella patens*^12^. No homologous sequences have been found in lower plant-related organisms whose genome is completely sequenced, such as *Chlamydomonas reinhardtii* or *Ostreococcus tauri*^13,14^. Annotation of WIP proteins revealed two domains. The N-terminal region contains degenerative sequence and two to three short conserved motifs with unknown function^5^. Reverse genetic experiment using TILLING showed that at least one of the conserved motifs is required for the proper function of the protein^15^. The C-terminal region is highly conserved and contains the zinc fingers and nuclear localization signals and is predicted to be implicated in DNA interaction.

In this study, we aimed to identify novel proteins that interact with CmWIP1 to help identify certain of the important regulatory networks involved in unisexual flower development. The yeast two-hybrid (Y2H) method is a powerful tool for high-throughput protein–protein interaction studies *in vivo*^16^. We generated a high-quality cDNA library from unisexual and hermaphrodite flowers and performed a yeast two-hybrid screen using CmWIP1 as bait. We showed that CmWIP1 physically interacts with a basic leucine zipper transcription factor, MELO3C022062_CmbZIP48, and that CmWIP1-CmbZIP48 protein interaction is mediated by the N-terminal part of CmWIP1. The bimolecular fluorescence complementation (BiFC) assays confirmed that CmWIP1 interacts *in planta* with CmbZIP48. As expected for a transcription factor, the interaction occurs in the nucleus. CmbZIP48 belongs to the S2 subclass of bZIP proteins. We further show that CmWIP1 interacts with MELO3C018916_CmbZIP42 another bZIP S2 subclass member. Thanks to the investigation of the spatiotemporal expression pattern in sex transition mutants, we demonstrated that the expression of the male promoting gene CmWIP1 correlates with the expression of CmbZIP48 in the carpel primordia of male flowers to lead to unisexual male flowers.

## Results and Discussion

### CmbZIP48 protein interacts with the N-terminal region of CmWIP1

**Figure 1:**
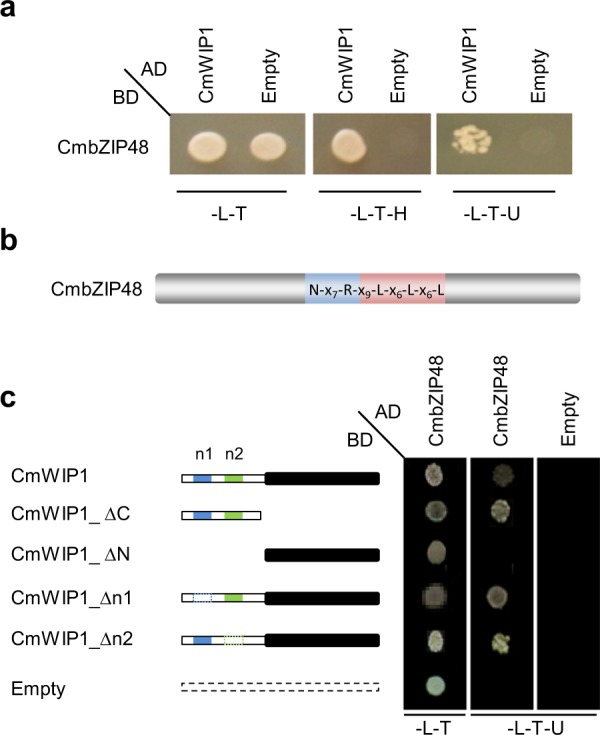
Identification of CmWIP1-CmbZIP48 protein interaction by yeast two hybrid screening. (**a**) Yeast cells transformed with CmWIP1-AD and CmbZIP48-BD growing on the selective medium. (**b**) Diagram of the bZIP domain of CmbZIP48. The basic region and the leucine zipper domain are shaded in blue and red, respectively. The sequence of the invariant residues within the bZIP domain of CmbZIP48 is given. (**c**) Diagram of the CmWIP1 deletion isoforms used for the Y2H interaction assays. The C terminal region of CmWIP1 is black filled. The conserved n1 and n2 domains are shaded in blue and green, respectively.

To identify the protein partners of CmWIP1, we used the yeast two-hybrid system (Y2H). Because CmWIP1 is expressed at early stages of the flower development, the cDNA library used as a prey to identify CmWIP1 partners was synthesized from RNA of male, female and hermaphrodite flower meristems at the stage where CmWIP1 is expressed. Out of the 3.5 10^6^ clones screened, 68 positive colonies were obtained after three times re-streaking on selective media. Sequencing and annotation of the positive clones revealed that 17 clones (25%) contain coding sequences of the *MELO3C022062* gene in-frame with the GAL4 Activation Domain (Fig. 1a). *MELO3C022062* is a single exon gene encoding a basic leucine zipper (bZIP) transcription factor of 179 amino acids, here after CmbZIP48. bZIP transcription factors are characterized by a conserved bZIP protein domain composed of two motifs, a basic region responsible for the DNA binding specificity and a leucine zipper required for protein dimerization (Fig. 1b)^17^. The bZIP transcription factors regulate crucial biological processes such as organ and tissue differentiation^18–20^, pathogen defence^21,22^, hormone and sugar signalling^23^, protection against biotic and abiotic stresses^24,25^ and flower and seed development^17,26^. CmbZIP48 shares 54% amino acid identity with AtbZIP42 (*AT3G30530*) and 50% identity with AtbZIP43 (*AT5G38800*).

To further analyse the regions involved in the CmbZIP48-CmWIP1 protein interaction, we generated CmWIP1 deletion mutants (Fig. 1c). To develop a logical approach for constructing CmWIP1 deletion mutants, we analyse the protein sequences of the six members of the WIP family in melon for candidate protein interaction motifs. This analysis revealed that melon WIP proteins can be divided in two domains, a non-conserved N-terminal half and a highly conserved C-terminal half starting with the three amino acids WIP and containing the four C_2_H_2_ zinc fingers. The observation that two conserved motifs are present within the non-conserved N-terminal half raises the possibility that these n1 and n2 subdomains may be involved in protein–protein interactions. Similar protein structure has also been reported for the *A*. *thaliana* WIP proteins^5^.

Based on the WIP protein domain conservation, we prepared different deletion mutants, such as CmWIP1_∆C (deletion of the C-terminal region) and CmWIP1_∆N (deletion of the N-terminal region). Interactions assays performed using these deletion mutants demonstrate that the C-terminal half of CmWIP1 is not required for the CmWIP1-CmbZIP48 interaction (Fig. 1c). By contrast the N-terminal region binds CmbZIP48 efficiently and is sufficient for this interaction (Fig. 1c). To map more precisely the subdomains necessary for the interaction with CmbZIP48 within the N-terminal region of CmWIP1, we generated CmWIP1_∆n1 and CmWIP1_∆n2 deletion mutants. We found that deletions of the conserved n1 or n2 domains had no effect on CmWIP1 interaction with CmbZIP48 (Fig. 1c). These results suggested that CmWIP1 interacts with CmbZIP48 through its non-conserved N-terminal regions.

### CmWIP1 interacts with CmbZIP48 *in planta*

**Figure 2:**
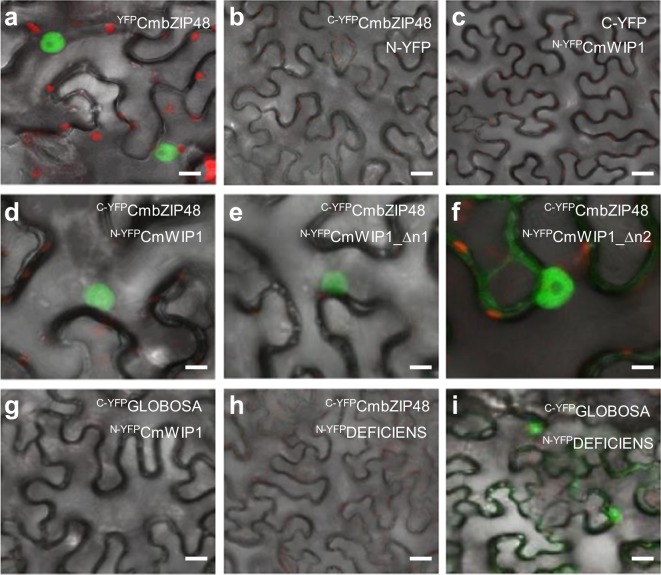
Nuclear *in planta* interaction of CmWIP1 and CmbZIP48 by BiFC. (**a**) The YFP fused at the N terminus of CmbZIP48 localized into the nucleus. (**b**–**i**) The BiFC experiments. The constructs combinations are indicated within each panel. Negative (**b**,**c**,**g**,**h**) and positive (**i**) controls are shown. Scale bars = 10 µm (**a**,**d**–**f**); 15 µm (**g**) and 30 µm (**b**,**c**,**h**).

Previous studies showed that all WIP proteins contain two monopartite nuclear localisation signals (NLS) located within the second and the fourth C2H2-ZnF domain and localize in the nucleus^5^. The CmbZIP48 is predicted to contain a monopartite NLS and is expected to localize in the nucleus. To characterize its subcellular localization, we fused the yellow fluorescent protein (YFP) at the N terminus of CmbZIP48 and showed that, as the WIP proteins, CmbZIP48 localized to the nucleus (Fig. 2a).

To address the physical interaction of CmbZIP48 and CmWIP1 *in planta*, we took advantage of the bimolecular fluorescence complementation (BiFC)^27,28^. We fused the C-terminal portion of the YFP (cYFP) to the full length CmbZIP48. The cYFP was fused to the N-terminus of CmbZIP48. For CmWIP1, we fused the N-terminal portion of YFP (nYFP) to the N-terminus of the full length CmWIP1. The expression of the cYFP-CmbZIP48 and nYFP-CmWIP1 fusions was driven by the constitutive CaMV 35S promoter. The constructs were agro-infiltrated into *Nicotiana benthamiana* leaves and CmbZIP48-CmWIP1 protein interaction was assayed based on the reconstitution of YFP fluorescence. Infiltration of cYFP-CmbZIP48 paired with nYFP or nYFP-CmWIP1 paired with cYFP yields no YFP fluorescence (Fig. 2b,c). In contrast, when cYFP-CmbZIP48 was assayed with nYFP-CmWIP1, a strong YFP signal was detected in the nucleus indicating that CmbZIP48 and CmWIP1 interact *in planta* and co-localize in the nucleus (Fig. 2d). Then, we assayed for *in planta* interaction between cYFP-CmbZIP and nYFP-CmWIP1_∆n1 or nYFP-CmWIP1_∆n2 truncated versions of CmWIP1 (Fig. 2e,f). As shown in yeast, the two CmWIP1 truncated versions were still able to interact with CmbZIP48 *in planta* in the nucleus (Fig. 2e,f).

To address the specificity of the CmbZIP48-CmWIP1 association, we assayed the interaction of CmWIP1 and CmbZIP48 with CmGLOBOSA and CmDEFICIENS, respectively. GLOBOSA and DEFICIENS are two nuclear MADS-box proteins known to interact in the nucleus (Fig. 2i)^29^. Fluorescent signal was detected neither for nYFP-CmWIP1 assayed with CmGLOBOSA (Fig. 2g) nor for cYFP-CmbZIP48 assayed with CmDEFICIENS (Fig. 2h), suggesting that the YFP signal observed when nYFP-CmWIP1 and cYFP-CmbZIP48 are co-agroinfiltrated results from the specific CmWIP1-CmbZIP48 protein interaction.

### Identification and classification of the bZIP transcription factors in the melon genome

To test whether other melon bZIP proteins could interact with CmWIP1, we performed an extensive genome-wide search using bZIP protein sequences from Arabidopsis, rice, tomato and cucumber as BLASTP queries against the melon genome database^30^. A total of 63 non-redundant CmbZIP transcription factors were identified (Supplementary Table 1). For convenience, we assigned a unique identifier to the *CmbZIPs* genes, *CmbZIP1* to *CmbZIP63*, based on their location on chromosomes 1 to 12 (Supplementary Fig. 1 and Supplementary Table 1). Three *CmbZIPs* mapped to unanchored scaffolds and were numbered *CmbZIP61*, *CmbZIP62* and *CmbZIP63* (Supplementary Fig. 1 and Table 1). The CmbZIP proteins range from 129 to 721 aa with an average size of 318 aa and with a range of molecular weights from 15.11 kDa to 78.67 kDa. The CmbZIP names and other details are summurized in Supplementary Table 1.

Compared to other plants, the number of *CmbZIP* genes and the sizes of CmbZIP proteins are comparable to the *bZIP* gene families of cucumber (64 bZIPs)^31^, medicago (65 bZIPs)^32^, chickpea (59 bZIPs)^32^, tomato (69 bZIPs)^33^ and Arabidopsis (77 bZIPs)^17^.

To analyse the evolutionary history the CmbZIP gene family, an unrooted phylogenetic tree was generated using a multiple sequence alignment of the CmbZIP proteins. The phylogenetic tree subdivided the melon bZIP genes family into 10 groups (A to I and S groups based on the Arabidopsis nomenclature in 17) with well-supported boostrap values (Supplementary Fig. 2). As expected, CmbZIP family members from the same group clustered together into the same clade (Supplementary Fig. 2). The two-species phylogenetic analysis of bZIPs among melon and Arabidopsis showed that all the clades contain melon and Arabidopsis bZIP proteins and no melon specific bZIP clade was observed (Supplementary Fig. 3). This interspecies tree also suggests the existence of homologous bZIP genes among melon and Arabidopsis and that melon bZIP proteins have been conserved during evolution.

The intron-exon organization of the members of a multigene family is an imprint of their evolution. To get a deeper insight into the evolution of the melon bZIP genes, we investigated the intron-exon structure of the 63 *CmbZIP* genes. Among all, we detected 15 (23%) intronless *CmbZIP* genes and most of them (14 genes) were clustered in the group S (Supplementary Fig. 2). Similar proportion of intronless *bZIP* genes has been reported in Arabidopsis, cucumber, tomato and legume plants. For the *CmbZIP* genes containing introns, the number of introns ranged from 1 to 11 and as expected *CmbZIP* genes from the same group tended to share similar gene structures (Supplementary Fig. 2). For example, the CmbZIP genes of group D and G contain 7 to 11 introns and 10 to 11 introns, respectively. Interestingly, all the CmbZIP genes of group C have 5 introns.

To physically map the CmbZIPs genes, we determined their chromosome locations (Supplementary Table 1). Among all, 3 CmbZIP genes were mapped on unanchored scaffolds and 60 CmbZIP genes were mapped on the 12 melon chromosomes and were distributed unevenly (Supplementary Fig. 1). Chromosomes 3, 1, 4 and 7 contain the largest numbers of CmbZIPs with 11, 9, 8 and 7 members, respectively, whereas chromosomes 2, 5, 9, and 12 contain only two members (Supplementary Fig. 1).

Tandem gene duplications are important mechanisms that drive the expansion of a gene family. In melon, we detected only four pairs of tandem duplication, CmbZIP6/7, CmbZIP29/30, CmbZIP34/35 and CmbZIP57/58, on chromosomes 1, 4, 6 and 11, respectively (Supplementary Table 1), suggesting a marginal contribution of tandem duplications to the CmbZIP gene family expansion.

### Two members of the CmbZIP S2 sub-group, CmbZIP48 and CmbZIP42, interact with CmWIP1

**Figure 3:**
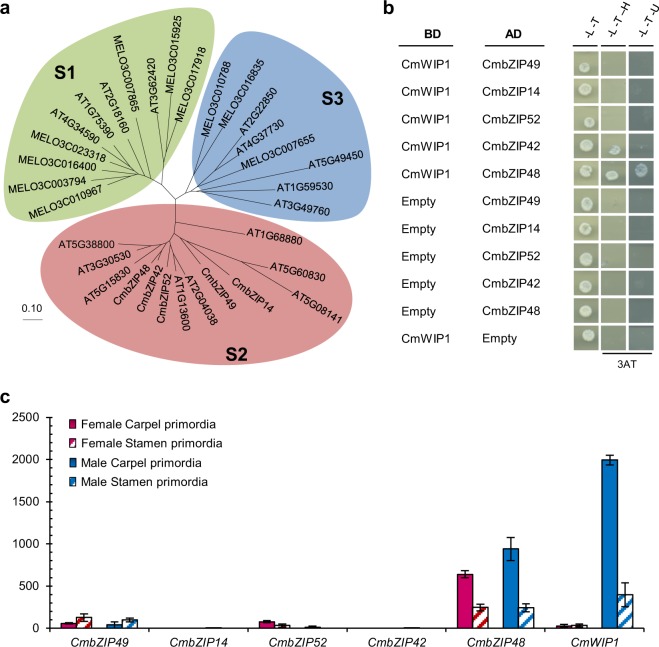
Targeted Y2H and expression pattern of the S2 CmbZIP. (**a**) Phylogenetic analysis of group S bZIP proteins in melon and Arabidopsis. The scale bar indicates 0.1 amino acid substitution per site. (**b**) Targeted Y2H assays between CmWIP1 and the five CmbZIP of the S2 sub-group. (**c**) Gene expression pattern of *CmWIP1* and the five S2 *CmbZIP* genes in the carpel and stamen primordia of female and male flowers of a monoecious plant.

In the Y2H and BiFC experiments, we identified and confirmed that CmbZIP48 interacts with CmWIP1 (Figs 1 and 2). *CmbZIP48* belongs to the group S of bZIP transcription factors (Supplementary Fig. 3 and Table 1). The phylogenetic analysis in melon and Arabidopsis revealed that the members of the group S can be divided into 3 sub-groups, S1, S2 and S3 and that *CmbZIP48* is part of the S2 sub-group (Fig. 3a). Global protein alignment shows that all the S2 bZIP proteins from melon and Arabidopsis share the typical bZIP protein domain, N-x_7_-R-x_9_-L-x_6_-L-x_6_-L (Supplementary Fig. 4).

To test whether other bZIP proteins of the S2 sub-group, here after, CmbZIP14, CmbZIP42, CmbZIP49 and CmbZIP52, could interact with CmWIP1, we used targeted Y2H assays (Fig. 3b). Yeast lines coexpressing CmbZIP14, CmbZIP49 or CmbZIP52 with CmWIP1 were unable to grow on selective media, suggesting no physical interactions (Fig. 3b). In contrast, yeast lines coexpressing CmbZIP42 and CmWIP1 were able to grow, as did yeast lines coexpressing CmWIP1 and CmbZIP48 (Fig. 3b). Interestingly, among the five melon S2 bZIP proteins, CmbZIP42 is the CmbZIP48-closest homolog, sharing 71% amino acid identity.

### CmWIP1 and CmbZIP48 are coexpressed in carpel primordia of male flowers

To weigh the biological significance of the physical interaction between two proteins, it is admitted that if the two interacting genes have also correlated expressions across various tissues, they are likely to control the same functions^34^. Thus, we compared the expression profiles of *CmWIP1* to *CmbZIP42* and *CmbZIP48* genes. For comparison we also analysed the expression profiles of CmbZIP14, CmbZIP49 and CmbZIP52, the three other S2 sub-group bZIP proteins that do not interact physically with CmWIP1.

Using *in situ* hybridization (ISH), we previously demonstrated that the spatial expression of *CmWIP1* is restricted to the carpel primordia of male flowers and no expression was detected in carpel primordia of buds determined to develop a carpel^15^. To obtain an in-depth resolution of the S2 *CmbZIP* spatial expression pattern, we isolated carpel and stamen primordia tissues from male and female flowers from a monoecious plant using laser capture microdissection (LCM). To check whether the quantitative real-time (qRT) gene expression analysis using microdissected tissues can be used as an alternative to the ISH, we first assayed the expression of *CmWIP1*. As for the ISH analysis, *CmWIP1* is mainly expressed in the carpel primordia of male flowers. Weak to no expression was detected in the stamen primordia of male flowers and carpel and stamen primordia of female flowers (Fig. 3c). This result demonstrate that the LCM-qRT gene expression analysis corroborate the results obtained using ISH experiment and thus can be used to determine the spatial gene expression pattern. Expression analysis of *CmbZIP14*, *CmbZIP49* and *CmbZIP52* showed weak to no expression in the carpel primordia of male flowers where *CmWIP1* is expressed (Fig. 3c). *CmbZIP42* was found not expressed in any sexual primordia of male or female flowers, explaining why the CmWIP1-CmbZIP42 protein interaction was not detected in the non-targeted Y2H screen (Fig. 3c). Interestingly, *CmbZIP48* was found mimicking the expression pattern of *CmWIP1* in male flowers, at the developmental stage where *CmWIP1* inhibits carpel development. Indeed, we found both genes highly expressed in the carpel primordia and weakly expressed in the stamina primordia of male flowers. In contrast, in female flowers, *CmbZIP48* is expressed in both carpel and stamen primordia, whereas *CmWIP1* is not expressed (Fig. 3c). Altogether, these results highlight that *CmWIP1* and *CmbZIP48* share a similar expression pattern and are spatially coexpressed in the stamen primordia of male flowers providing the plant organ context for the CmWIP1-CmbZIP48 protein interaction.

### Integration of the *CmbZIP48* in the sex determination model

We previously demonstrated that sex determination in melon relies on the interplay between alleles of three sex determination genes, *M*, *G* and *A*. In monoecious plants, male flowers results from non-expression of *CmACS11*, that permits *CmWIP1* expression. Female flowers develop on at the youngest nodes of the growing vines expressing *CmACS11*, which represses the expression of *CmWIP1*, and thus, releasing the expression of *CmACS-7* inhibiting stamina development. If nonfunctional *CmACS-7* is expressed, hermaphrodite, instead of female, flowers develop. Androecious plants result from a loss-of-function of *CmACS11* leading to expression of *CmWIP1* in all flowers on a plant. Gynoecious plants are obtained by inactivation of *CmWIP1* function and hermaphrodite plants are obtained by inactivation of *CmWIP1* and *CmACS-7*^3,6,15^.

**Figure 4:**
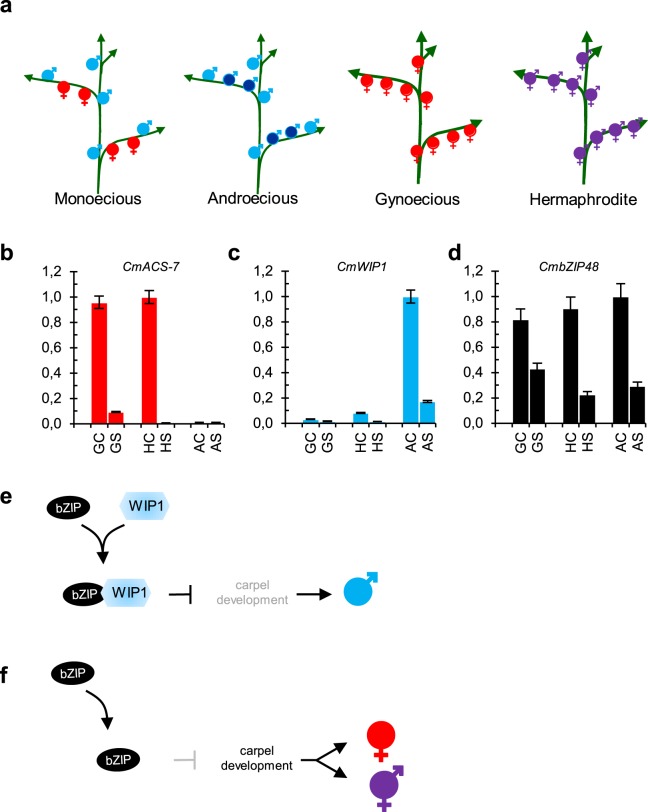
Genetic model integrating CmWIP1 and bZIP48 interaction. (**a**) Schematic representation of the monoecious, androecious, gynoecious and hermaphrodite sexual morphs in melon. (**b**–**d**) Quantitative RT-PCR of *CmACS-7*, *CmWIP1* and *CmbZIP48* in carpel and stamen primordia of female, hermaphrodite and male flowers of gynoecious, hermaphrodite and androecious melons, respectively. Shown are the mean + − SD of three biological replicates. GC: gynoecious carpel primordia, GS: gynoecious stamen primordia, HC: hermaphrodite carpel primordia, HS: hermaphrodite stamen primordia, AC: androecious carpel primordia, AS: androecious stamen primordia. (**e**,**f**) Model of the sex-determination pathway in melon integrating the CmWIP1-CmbZIP48 co-expression and protein interaction. (**e**) When *CmWIP1* and *CmbZIP48* are coexpressed, CmWIP1-CmbZIP48 complex forms and represses carpel development leading to the rise of a male flower. (**f**) When *CmWIP1* is not expressed, the expression of the genes involved in carpel development leads to a female flower.

To integrate the role of the CmWIP1-CmbZIP48 interaction in our sex determination model, we investigated the expression of *CmACS-7*, *CmWIP1* and *CmbZIP48* genes in flowers of androecious, gynoecious and hermaphrodite melon mutants (Fig. 4a). As expected, *CmACS-7* is highly expressed in the carpel primordia of female and hermaphrodite carpel-bearing flowers whereas weak to no *CmACS-7* expression was detected in the stamen primordia of female and hermaphrodite flowers and carpel and stamen primordia of male flowers (Fig. 4b). *CmWIP1* was found highly expressed in the carpel primordia of male flowers. In the female and hermaphrodite flowers, *CmWIP1* was not expressed (Fig. 4c). *CmbZIP48* expression was not affected by the flower sexual type and was found strongly expressed in the carpel primordia of female, hermaphrodite and male flowers (Fig. 4d).

Altogether, these data support a model in which the coexpression and protein-protein interaction of CmWIP1 and CmbZIP48 turn on the carpel primordia abortion leading the development of a male flower. The lack of *CmWIP1* expression yields carpel development and leads to the raising of a female flower (Fig. 4e,f). This model also suggests that *CmbZIP48* expression is independent of *CmWIP1* expression, indicating that *CmbZIP48* may recruit other transcription factors required for carpel development. In Arabidopsis, the bZIP transcription factor HY5 was identified as a negative regulator of ethylene biosynthesis. HY5 activates the expression of the transcriptional repressor *AtERF11* which further represses the expression of *AtACS2* and *AtACS5*^35^. This may suggest that CmWIP1-CmbZIP48 complex recruit a melon *ERF* gene to repress the expression of *ACS* genes. In this scenario, the repression of *CmACS-7* leads to the stamina development and the repression of *CmACS11* maintain *CmWIP1* expression that blocks the development of the carpel thus yielding a male flower. Furthermore, the integration of the *CmbZIP48* into the genetic model of sex determination sheds light on the molecular mechanisms of how *CmWIP1* inhibits the carpel development.

## Methods

### The yeast-two hybrid system

The standardized yeast-two hybrid protocol^36^ was used to perform the screen in which CmWIP1 protein served as bait and cDNA library as a prey in order to identify interacting protein partners of CmWIP1. Total RNA was extracted from male, female and hermaphrodite flowers just before and after the arrest of the inappropriate organ^37^ using the Trizol reagent (Invitrogen). The cDNA library was synthesized from 6 µg of total RNA using the cDNA Library Construction Kit (Invitrogen). The CmWIP1 bait gene was fused to the GAL4 DNA binding domain into pDEST^TM^32 vector. The cDNA library was fused to the GAL4 transcriptional activation domain cloned into the pDEST^TM^22 vector. For the two-hybrid assays, yeast cells MaV203 (Invitrogen) were cotransformed with the two constructs according to the protocol of Dohmen *et al*.^38^. The ability to drive the *HIS3* reporter gene was assessed by growing transformants on selective medium lacking tryptophan, leucine, and histidine and supplemented with 60 mM 3-amino-19,29,49-triazole (3-AT). Handling of yeast cultures and plate growth assays were performed as described in the Yeast Protocols Handbook (Clontech).

### Bimolecular fluorescence complementation (BiFC)

Visualization of the interaction between two proteins of interest in living *Nicotiana benthamiana* plant cells was achieved through the utilization of standard BiFC protocols^39^. The coding sequence of the genes of interest were cloned into the vectors pBiFC2 (YFP^N^), and pBiFC3 (YFP^C^). The two target proteins fused to the YFP fragment, either YFP^N^ or YFP^C^, were transiently expressed in leaves of 3-week-old *N*.*benthamiana* plants by infiltration as described in Voinnet *et al*.^40^. Upon interaction between the two target proteins, the YFP^N^ and YFP^C^ fragments restore fluorescence. YFP fluorescence was detected 3 days after infiltration by using the 514-nm laser line of a LSM 710 confocal laser scanning microscope (Carl Zeiss) equipped with an argon laser. Fluorescent images of interactions or absence of fluorescence in tobacco leaf cells were captured using the ZEN software.

### Bioinformatic and phylogenetic analysis

Multiple sequence alignment of the S2 bZIP protein sequences of melon and Arabidopsis was performed using the ClustalW (http://www.ebi.ac.uk/Tools/clustalw)^2^. Phylogenetic trees were constructed using the Neighbor-Joining method by MEGA 7.0 (http://www.megasoftware.net/index.html).

### Identification of bZIP proteins in melon

To identify candidate bZIP proteins from melon, the melon database (http://www.melonomics.net/) was searched first using the keywords ‘bZIP’. In addition, Arabidopsis, rice, tomato and cucumber bZIP protein sequences were downloaded from The Arabidopsis Information Resource (http://www.arabidopsis.org/), the Rice Genome Annotation Project (http://rice.plantbiology.msu.edu/), the Sol Genomics Network (http://solgenomics.net/) and the Cucurbit Genomics Database (http://cucurbitgenomics.org/), respectively. These sequences were used to identify homologous peptides from melon by performing a BLASTP search at melon genome v3.5 database (http://www.melonomics.net/)^30^. The BLAST E-value was set to 1e^−^3. The retrieved sequences were searched using SMART (http://smart.embl-heidelberg.de/), and Pfam (http://pfam.sanger.ac.uk/) databases for the presence of the conserved bZIP domain. Finally, repeated and incomplete sequences were removed manually and the non-redundant CmbZIP sequences were subjected to further analyses.

### Chromosomal distribution and gene structure

The CmbZIP genes were mapped onto the corresponding chromosomes by BLASTP against the melon genome v3.5 database (http://www.melonomics.net/) using default settings. The CmbZIP genes were plotted onto the melon chromosomes according to their ascending physical position (bp). Tandem duplications were characterized as adjacent genes of same sub-family located within 10 predicted genes apart or within 30 kbp of each other.

The exon-intron structure of the CmbZIP genes was determined using the Gene-Structure Display Server (gsds.cbi.pku.edu.cn) through comparison of their coding sequence (CDS) with their corresponding genomic sequence.

### Laser capture microdissection

#### Tissue embedding

Flowers buds were fixed in RCL2 (Excilone) with 0.01% triton, vacuum 4 times for 15 min and kept in the fixative overnight at 4 °C. Tissues were dehydrated at 4 °C in a graded series of ethanol (70% for 30 min, 96% for 30 min, 100% for 3 × 30 min), followed by a graded series of ethanol:histoclear bath (3:1, 1:1, 1:3 for 1 h each). Histoclear was then substituted by Surgipath Paraplast plus tissue embedding media (Leica Biosystems) overnight at 60 °C. Finally, flowers were poured into paraffin blocks, cooled and stored at −20 °C.

#### Laser-assisted microdissection

Longitudinal flowers sections of 8 µm were cut using a Rotary microtome (HM 3555 Microtom). Ribbons were stretched on UV-treated, 1 mm PEN-membrane covered slides (Arcturus Bioscience, Excilone). Each slide corresponding to 15–25 sections of flowers at bisexual stage. Side were de-paraffined, and laser capture microdissection was immediately conducted with a Palm DIC FLUO Microdissection System (Zeiss). The contour of stamen or carpel primordia were cut with the laser and target regions were automatically catapulted into Adhesive cap 500 clear (Zeiss).

#### RNA extraction

Cells were lysed immediately after dissection, using the PicoPure® RNA Isolation Kit (Arcturus Bioscience, Excilone) and stored at −20 °C before RNA extraction. RNA quality and concentration were evaluated with a Bioanalyser 2100 (Agilent Technologies) on Agilent RNA Pico chips. RNA recovery was from 500–1000 pg/µl, with a RIN at 7.

#### Preparation of cDNA libraries

2 ng of total RNA was used for each cDNA library preparation using the SMARTer Ultra Low RNA Kit for Illumina Sequencing from Clontech according to manufacturer’s instructions (16 cycles of PCR were used for cDNA amplification). cDNA were used to assessed the expression of the *S2 bZIP* and *CmWIP1* genes by qPCR experiments.

### Reverse transcription polymerase chain reaction (RT-PCR) and qPCR

Total RNA was extracted from frozen flowers with the Trizol reagent (Invitrogen). Contaminating DNA was removed by DNaseI treatment (Invitrogen). First strand cDNA was synthesized from 2 μg of total RNA with the Superscript® III reverse transcriptase (Invitrogen). Primer design was performed with the Primer3 software (http://frodo.wi.mit.edu/cgi-bin/primer3/primer3_www.cgi). Primers sequences are listed in Supplementary Table 2. To check specificity of the designed primers, all amplicons were sequenced and blasted against NCBI database. Polymerase chain reactions were performed in an optical 384-well plate with the Bio-Rad CFX96 Real-time PCR apparatus, with qPCR MasterMix Plus for SYBR® Green I w/o ROX (Eurogentec) and according to manufacturer’s instructions. PCR amplification specificity was verified by a dissociation curve (55 °C to 95 °C). A negative control without cDNA, technical replicates on three independent synthesis of cDNA (derived from the same RNA sample), and three independent biological experiments were performed in all cases. To compare data from different PCR runs and cDNA samples, CT values for *CmbZIP49*, *CmbZIP14*, *CmbZIP52*, *CmbZIP42*, *CmbZIP48* and *CmWIP1* were normalized to the CT value of *CmActin2* (primers shown in Supplementary Table 2). The gene relative expressions were determined as described in^6^.

## Supplementary information

Supplementary FIgures

## Data Availability

All the data supporting the results of this paper are present in the paper and/or the supplementary materials. All relevant data are available from the corresponding author on request.
