# Supplementary material for: The gynoecious CmWIP1 transcription factor interacts with CmbZIP48 to inhibit carpel development
Source: Sci Rep. 2019 Oct 28;9:15443. doi: 10.1038/s41598-019-52004-z (PMC6817838; doi:10.1038/s41598-019-52004-z)
Supplement: Supplementary file 1 — Supplementary FIgures [file 41598_2019_52004_MOESM1_ESM.pdf]

Supplementary information

The gynoecious CmWIP1 transcription factor interacts with CmbZIP48 to inhibit carpel development.

John SY. Eleblu[1], Aimen Haraghi[1], Brahim Mania[1], Celine Camps[1], Dali Rashid[1], Halima Morin[1], Catherine Dogimont[2], Adnane Boualem[1,3] and Abdelhafid Bendahmane[1,3,*]

[1]Institute of Plant Sciences Paris-Saclay IPS2, CNRS, INRA, University Paris-Sud, University of Evry, University Paris-Diderot, University of Paris-Saclay, Orsay cedex, France.

[2]UR 1052, Unité de Génétique et d'Amélioration des Fruits et Légumes, INRA, BP94, Montfavet, France.

[3]These authors jointly supervised this work: Abdelhafid Bendahmane and Adnane Boualem

*Corresponding author. E-mail: abdelhafid.bendahmane@inra.fr

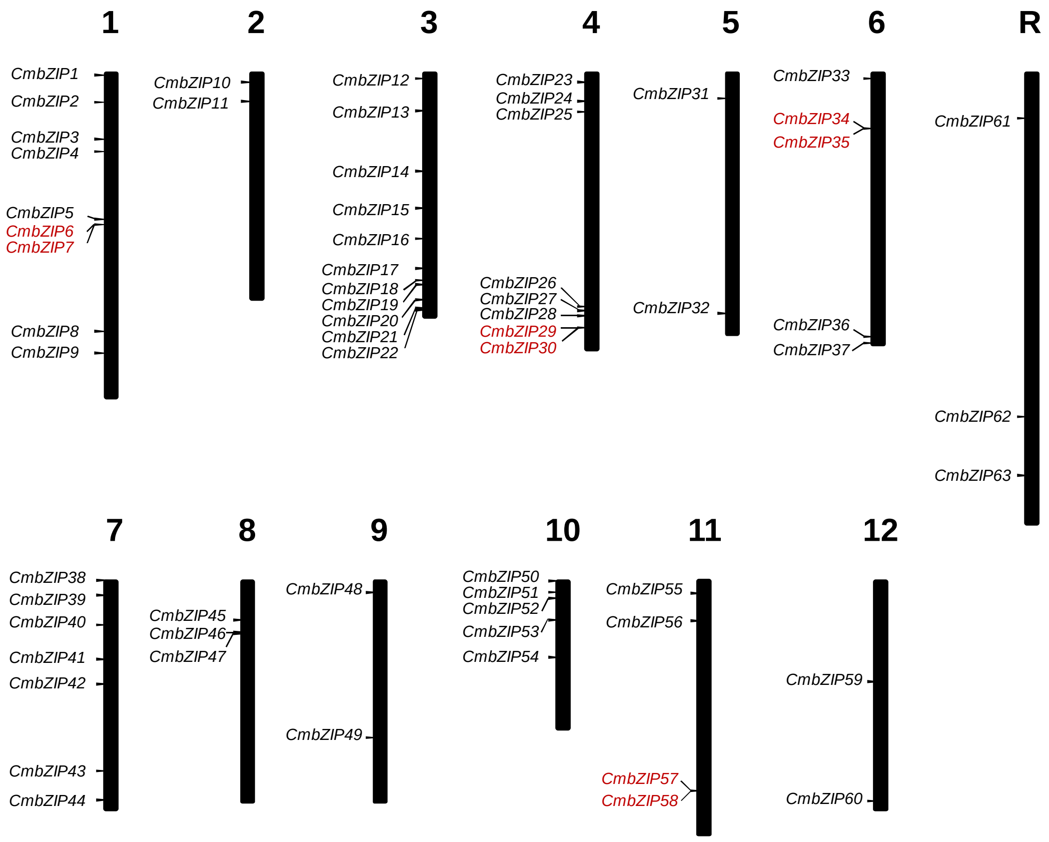

Supplementary Figure 1: Distribution of *CmbZIP* genes on melon chromosomes.

The scaled representation shows the physical locations of the *CmbZIPs* genes on the 12 melon chromosomes. Unanchored scaffolds are assembled in a random chromosome named R. Tandem-duplicated genes are indicated in dark red.

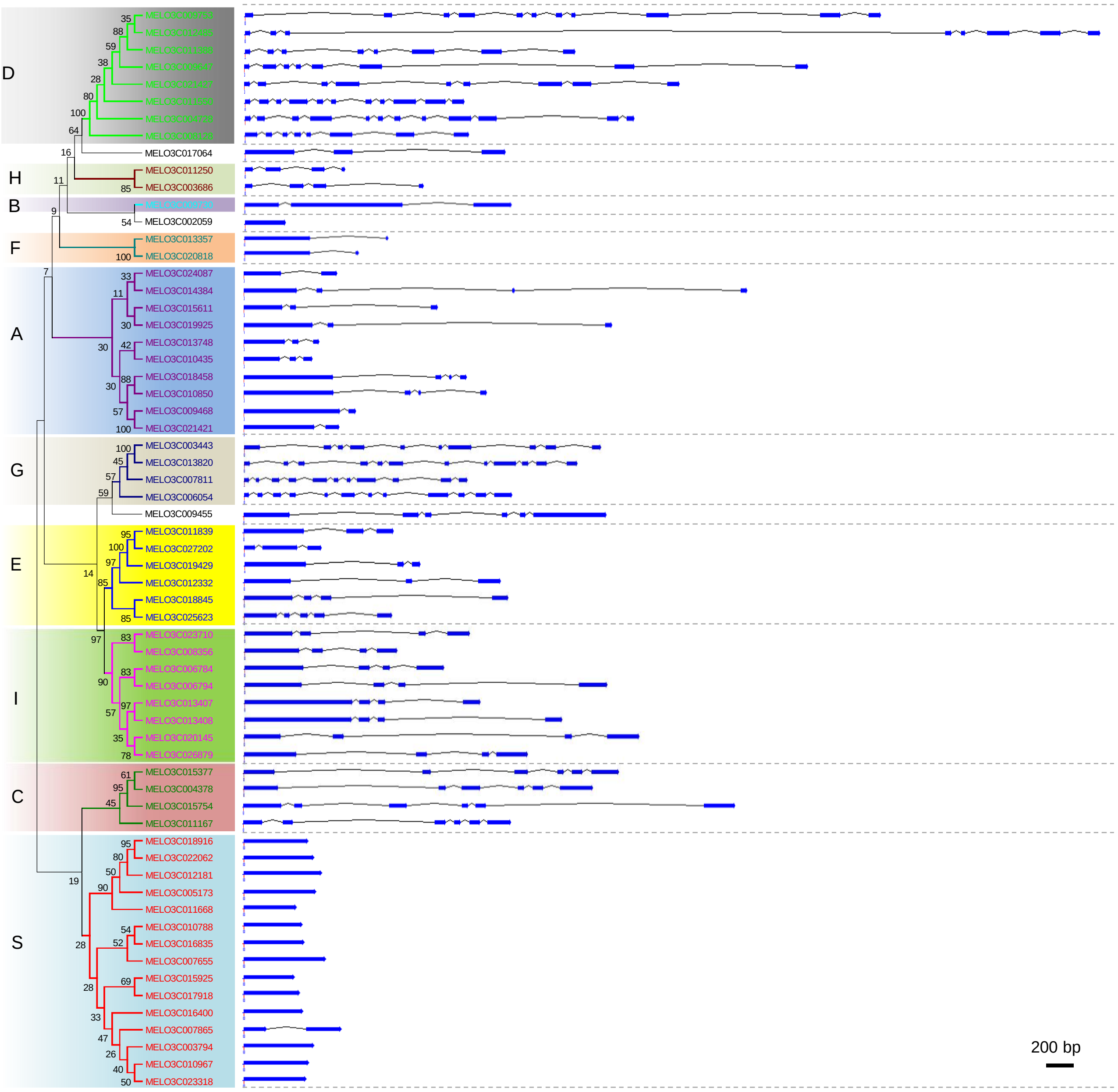

Supplementary Figure 2: Phylogenetic analysis and exon-intron structures of the melon CmbZIP genes.

(**Left**) Unrooted phylogenetic tree of the 64 CmbZIP proteins. Numbers on the tree branches indicated bootstrap values. (**Right**) Exon-intron structure of the melon CmbZIP genes. Blue boxes represent exons and black lines represent introns.

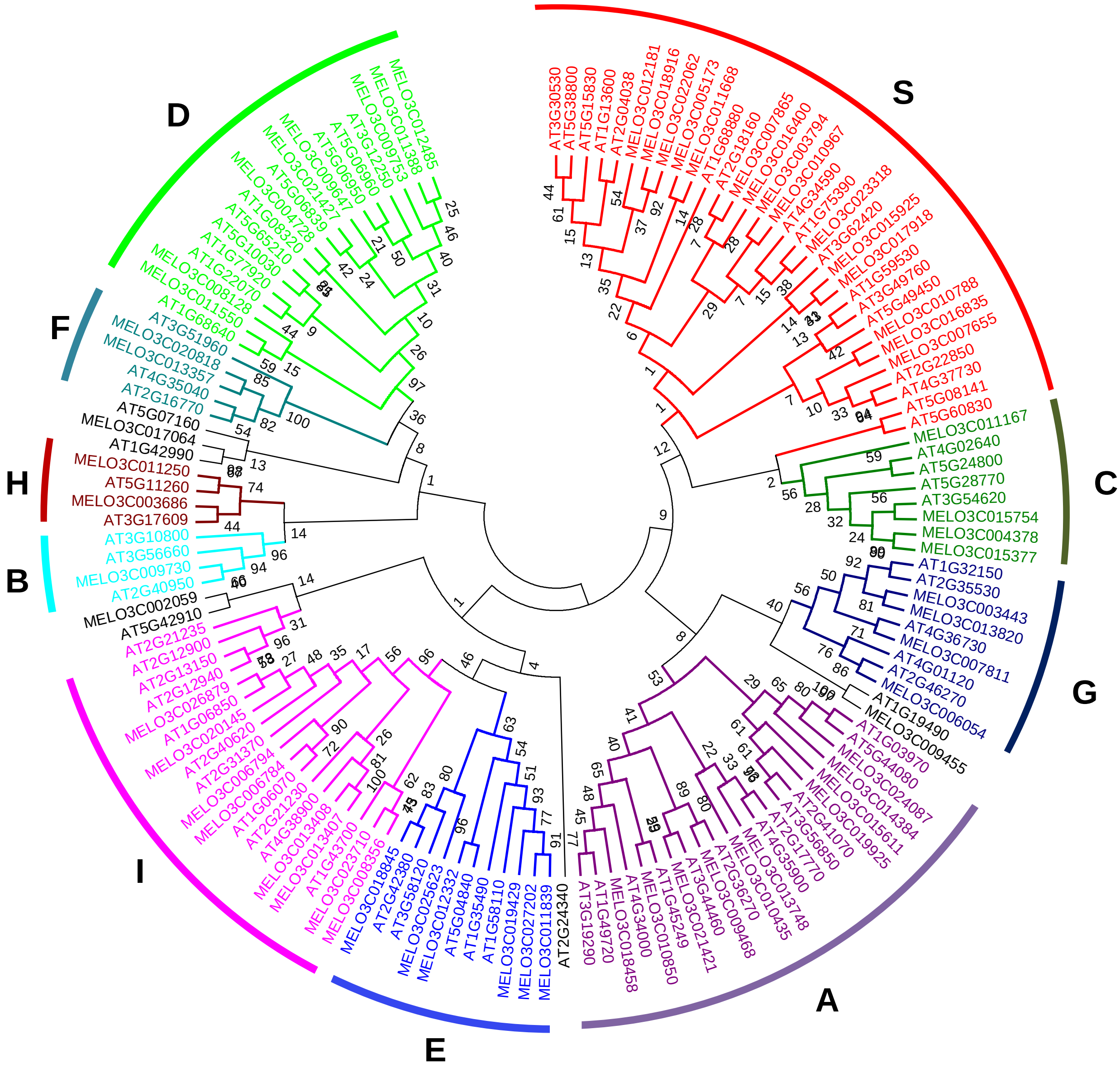

Supplementary Figure 3: Phylogenetic tree of melon and Arabidopsis bZIP proteins. The bZIP protein sequences were aligned by CLUSTALW and the phylogenetic tree was constructed using the Neighbor-Joining method by MEGA 7.0. Boostrap values from 1000 replicates are indicated in each node. The CmbZIP proteins are grouped into 10 groups (A-I and S).

```
MELO3C011668_CmbZIP14    ---ADERKQRRMISNRESARRSRMRKQKHVKELCSQLLRLCTQKHELEEKLRVLMESQQRL
MELO3C018916_CmbZIP42    LSLINERKQRRMISNRESARRSRMRKQKHLDELWSQVLWLRNENHQLIDKLNQVSECHDRA
MELO3C005173_CmbZIP49    RIVVDERKQRRMISNRESARRSRMRKQKHLDELWSVVVRLRTENHSLMEKLNQLTDSEQQL
MELO3C022062_CmbZIP48    QSLINERKQRRMISNRESARRSRMRKQKHLDELWSQVLWLRNENHQLIDKLNQVSDCHDKV
MELO3C012181_CmbZIP52    KSIIDERKQRRMISNRESARRSRMRKQKHLDELWSQVLRLRTENHKLIDKLNHVSDNHEKV
AT3G30530_AtbZIP42       NNIINERKQRRMISNRESARRSRMRKQRHLDELWSQVMWLRIENHQLLDKLNNLSESHDKV
AT1G13600_AtbZIP58       -MVIDERKQRRMISNRESARRSRMRKQRHLDELWSQVIRLRTDNHCLMDKLNRVSESHELA
AT2G04038_AtbZIP48       -MVLDERKQRRMLSNRESARRSRMRKQRHLDELWSQVIRLRNENNCLIDKLNRVSETQNCV
AT5G38800_AtbZIP43       KEIINERKQKRKISNRESARRSRMRKQRQVDELWSQVMWLRDENHQLLRKLNCVLESQEKV
AT5G15830_AtbZIP3        IFVINERKQRRMVSNRESARRSRMRKQRHLDELLSQVAWLRSENHQLLDKLNQVSDNNDLV
AT5G08141_AtbZIP75       ----EEKRLRRMASNRESARRTRMRERMMKEGLQMQVKQLMAYNQFLYNKYISLLEYNHQI
AT5G60830_AtbZIP70       GLAPEERRARRMVSNRESARRSRMRKKKQIEELQQQVEQLMMLNHHLSEKVINLLESNHQI
AT1G68880_AtbZIP8        ----NERKRRRKVSNRESARRSRMRKQRHMEELWSMLVQLINKNKSLVDELSQARECYEKV
```

Supplementary Figure 4: Protein sequence alignment of the conserved bZIP domains of S2 sub-group proteins.

Sequences were aligned using ClustalW. Conserved amino acids are highlighted in black. Invariant residues within the bZIP domain are colored in red.
